# Invasive Listeriosis of Intracardiac Device

**DOI:** 10.1155/2018/1309037

**Published:** 2018-04-17

**Authors:** Prerna Sharma, Mohamed Yassin

**Affiliations:** ^1^Internal Medicine Resident, UPMC Mercy, Pittsburgh, PA, USA; ^2^Infectious Diseases, UPMC Mercy, Pittsburgh, PA, USA

## Abstract

**Introduction:**

*Listeria monocytogenes* is a food-borne pathogen which can cause invasive infection in immunocompromised adults. Listeria has been known to cause infections during pregnancy and in older adults. Listeria endocarditis is a rare condition. A case of listeria-related intracardiac device infection is reported below.

**Case Report:**

A 74-year-old male with a past medical history of coronary artery disease, congestive cardiac failure, permanent atrial fibrillation status after nodal ablation, and placement of a biventricular pacemaker presented to the hospital with complaints of generalized fatigue. He was found to have listeria bacteremia, and transthoracic echocardiogram (TTE) showed pacemaker lead vegetation. The patient was treated with 6 weeks of vancomycin followed by oral suppression with amoxicillin.

**Discussion:**

Listeria can affect native valves, prosthetic valves, or nonvalvular intracardiac devices. The mean age of prosthetic valve endocarditis has been reported to be 67 years with male-to-female ratio 1.7 : 1 and mitral-to-aortic valve ratio 1.3 : 1. There have been case reports of listeria prosthetic valve endocarditis; however, there is paucity of literature on listeria-related pacemaker lead infection. Treatment is mostly a combination of penicillin and aminoglycosides for 4–6 weeks. Surgical removal of the infected device is preferred. Invasive listeriosis is a rare but fatal entity which should be identified and treated promptly to ensure a good outcome.

## 1. Introduction

*Listeria monocytogenes* is a facultative intracellular, Gram-positive aerobic rod which is mostly transmitted from contaminated food and can cause benign diarrheal illness in immunocompetent individuals [1]. It is a food-borne pathogen spread through consumption of contaminated food like dairy (raw unpasteurized milk or eggs, soft cheese), poultry, fish, shellfish, and so on [2]. Maternal infections during pregnancy are known to cause miscarriages, preterm labor, and neonatal meningitis. Invasive listeriosis can occur in immunocompromised patients manifesting as meningitis or bacteremia/septicemia [1]. We report a case of invasive listeriosis and pacemaker lead infection treated with vancomycin.

## 2. Case Report

A 74-year-old male with a past medical history of coronary artery disease, congestive cardiac failure, permanent atrial fibrillation status-post nodal ablation, and placement of a biventricular pacemaker presented to the hospital with complaints of generalized fatigue. The patient denied other constitutional symptoms including chest pain, dyspnea, abdominal pain, diarrhea, or dysuria. Vitals were as follows: afebrile, blood pressure 116/80, pulse 72, respiratory rate 21, and saturating well on 3 litres/minute nasal cannula. Physical examination showed pitting edema in bilateral lower extremities till knees, decreased bibasilar breath sounds, and jugular venous distension. There was no rash, painful nodes or splinter hemorrhages. Holosystolic murmur was heard at the apex, JVD ∼18 cm H_2_O. Laboratory investigations showed normal WBC count, neutrophilia with 17 bands, 26 lymphocytes, and 8 monocytes. CRP was elevated at 4.3. The patient also had a troponin level of 0.22; however, this was found to be chronically elevated. Chest X-ray showed cardiomegaly, but no pulmonary vascular congestion, consolidation, nor effusions. Urinalysis did not show any evidence of infection. Overnight, the patient developed fever with a maximum temperature of 39.2°C.

Blood cultures turned positive for Gram-positive rods at which time the patient was started on vancomycin. Echocardiogram was performed due to concerns of endocarditis and showed left ventricular ejection fraction 20–25%, severe diffuse hypokinesis, moderate mitral and tricuspid regurgitation, and a 1 mm mobile strand in the left atrium attached to pacer lead concerning vegetation. Final blood cultures revealed *Listeria monocytogenes*, and two sets of subsequent cultures grew listeria. TEE was not performed as it would not have changed management. The patient was not a surgical candidate for pacemaker removal due to comorbidities and advanced heart failure. Antibiotic therapy with vancomycin was continued. Ampicillin was not used due to high sodium content in the infusion in setting of decreased systolic function. The patient received 6 weeks of IV vancomycin followed by oral suppression with amoxicillin. There was appropriate clinical response to the abovementioned treatment with resolution of fever and subjective improvement.

## 3. Discussion

Invasive listeriosis has been reported in immunocompromised adults. The ability to cause invasive infection is largely dependent on host factors as well as bacteria's capacity to cross the intestinal barrier [2]. The mechanism of bacterial inoculation in our patient is unclear. The CDC reported a case fatality rate of 21% out of 1651 cases reported nationwide from 2009 to 2011 with an average incidence of 0.29 cases per 100,000 populations. Fifty-eight percent of cases were >65 years and 14% infections were pregnancy-related. Most of the cases not associated with pregnancy were <65 years, and they had underlying comorbidities such as malignancy, immunosuppressive therapy, diabetes, renal failure, congestive heart failure, cirrhosis, alcohol abuse, and HIV/AIDS [3].

Listeria endocarditis is a rare condition which can affect native valves, prosthetic valves [4, 5], or nonvalvular intracardiac devices [6]. A review of 68 patients with listeria endocarditis from 1955 to 2000 showed that most patients were immunosuppressed and did not have a documented exposure to contaminated food. Patients with native valve endocarditis had a higher mortality rate compared to those with prosthetic valve endocarditis [5].

Prosthetic valve endocarditis may present with new valvular regurgitation, dehiscence of prosthetic valve, and intracardiac mass of paravalvular abscess [4, 7, 8]. Best et al. reviewed 30 cases of listeria prosthetic valve endocarditis and reported a mean age of 67 years with male-to-female ratio 1.7 : 1 and mitral-to-aortic valve ratio 1.3 : 1. The most common clinical features were fever (89%), new murmur (74%), and atrial fibrillation (37%) [4]. Surgical intervention is warranted in patients with valve perforation or rupture, acute valvular dysfunction with heart failure, anterior mitral leaflet >10 mm in size, embolic events on antibiotic therapy, and so on [7].

Cardiac device-related infections can present with signs and symptoms similar to infective endocarditis (endovascular portion of device is involved) or as local infection if the device is implanted subcutaneously [9]. The reported rate of pacemaker infections is 0.13% to 19.9% and vegetation can be found on the tricuspid valve or anywhere along the course of electrode including the endocardium of the right atrium/ventricle [9].

Blood cultures or surgical cultures (valve) along with echocardiographic findings are diagnostic; however, bacterial gene PCR has been shown to be useful in culture-negative listeriosis [10]. Treatment consists of antimicrobials, with or without surgery. However, removal of the device is preferred due to high rate of relapsing bacteremia in spite of antibiotic therapy [9]. Surgical intervention in the abovementioned case was deferred due to high surgical risk. Penicillin and gentamicin combination for 4–6 weeks can be used; other treatment options include vancomycin or linezolid [8, 10]. Patients with penicillin allergy can be treated with trimethoprim-sulfamethoxazole or erythromycin [1]. Our patient was treated with vancomycin and long-term suppression with oral amoxicillin.

## 4. Conclusion

There have been case reports of listeria prosthetic valve endocarditis; however, there is paucity of literature on listeria-related pacemaker lead infection. The treatment is mostly with beta-lactams; however, it is varied on case-by-case basis. Invasive listeriosis is a rare but fatal entity and should be treated promptly to ensure a good outcome.
